# Quantification of C_60_-induced membrane disruption using a quartz crystal microbalance†

**DOI:** 10.1039/c7ra13690k

**Published:** 2018-03-09

**Authors:** Yuxuan Zeng, Qi Wang, Qiu Zhang, Wei Jiang

**Affiliations:** Environment Research Institute, Shandong University Jinan 250100 China jiangw@sdu.edu.cn +86-531-88361990 +86-531-88366072; School of Chemistry and Chemical Engineering, Shandong University Jinan 250100 China

## Abstract

Direct contact between fullerene C_60_ nanoparticles (NPs) and cell membranes is one of mechanisms for its cytotoxicity. In this study, the influence of C_60_ NPs on lipid membranes was investigated. Giant unilamellar vesicles (GUVs) were used as model cell membranes to observe the membrane disruption after C_60_ exposure. C_60_ NPs disrupted the positively charged GUVs but not the negatively charged vesicles, confirming the role of electrostatic forces. To quantify the C_60_ adhesion on membrane and the induced membrane disruption, a supported lipid bilayer (SLB) and a layer of small unilamellar vesicles (SUVs) were used to cover the sensor of a quartz crystal microbalance (QCM). The mass change on the SLB (Δ*m*_SLB_) was caused by the C_60_ adhesion on the membrane, while the mass change on the SUV layer (Δ*m*_SUV_) was the combined result of C_60_ adhesion (mass increase) and SUV disruption (mass loss). The surface area of SLB (*A*_SLB_) was much smaller than the surface area of SUV (*A*_SUV_), but Δ*m*_SLB_ was larger than Δ*m*_SUV_ after C_60_ deposition, indicating that C_60_ NPs caused remarkable membrane disruption. Therefore a new method was built to quantify the degree of NP-induced membrane disruption using the values of Δ*m*_SUV_/Δ*m*_SLB_ and *A*_SUV_/*A*_SLB_. In this way, C_60_ can be compared with other types of NPs to know which one causes more serious membrane disruption. In addition, C_60_ NPs caused negligible change in the membrane phase, indicating that membrane gelation was not the mechanism of cytotoxicity for C_60_ NPs. This study provides important information to predict the environmental hazard presented by fullerene NPs and to evaluate the degree of membrane damage caused by different NPs.

## Abstract 2

Fullerene C_60_ NPs adhere on lipid membrane due to electrostatic force and cause membrane disruption.

## Graphical abstract

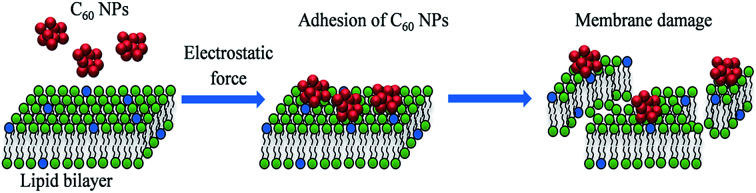

## 1. Introduction

Since its discovery in 1985, fullerene C_60_ has attracted great attention because of its unique spherical cage-like molecule structure.^1^ The extraordinary properties of C_60_ and its derivatives possibly cause them be employed in optical, biological and electronic engineering fields.^2–4^ Large amounts of C_60_ its derivatives are possibly released into the environment, hence their biological toxicities and environmental impact must be considered. C_60_ and its derivatives have been reported to cause toxicities on mammalian cell lines, bacteria, invertebrates and fish.^5–9^ The direct contact between nanoparticles (NPs) and cell membranes is suggested to cause cytotoxicity.^10^ Therefore both computer simulations^11–16^ and experimental studies^15,17–19^ have been conducted to investigate the interaction between C_60_ and cell membranes. C_60_ possibly disturbs normal cellular functions *via* lipid peroxidation, production of reactive oxygen species,^17,18^ membrane conformational changes,^11^ and membrane disruption.^19^

The adhesion of NPs on membrane and the subsequent influence on the structure of lipid membranes is a possible cytotoxic mechanism. Hence, the membrane damage caused by different types of NPs should be compared, which is important to select or design safe nanomaterials. A reliable experimental method is required to quantify the C_60_ adhesion on membrane and the induced membrane damage. The living cell is a complicated and dynamic system. For such a purpose, artificial lipid membranes are more convenient and reproducible models to avoid the uncertainty from living cells.^20^ The limitation of artificial membranes is that they do not contain all the lipid and protein components as the plasma cell membrane. Those complicated contaminants of lipids and proteins perform various functions and interact differently with nanoparticles,^21^ which cannot be mimicked by any single type of artificial membranes. However, artificial membranes are flexible to be modified according to the research purpose. Giant unilamellar vesicles (GUVs) are good models to observe the membrane disruption and morphologic change directly.^22,23^ Membrane charge and components are convenient to be adjusted in GUVs to figure out the interaction mechanism. The membrane disruption is possibly quantified *via* intact vesicle counting,^24^ but it is time-consuming and the accuracy depends on the number of counted vesicles and the observer's experiences. Moreover, the mass of adhered NPs on membrane cannot be monitored by the above-mentioned methods. New techniques are still needed to meet the quantification requirements.

Quartz crystal microbalance with dissipation monitoring (QCM-D) has been used to investigate the interaction of NPs with bio-surface.^25–27^ By the real-time monitoring of the crystal frequency shifts, the mass of adhered NPs on membrane-coated crystal sensor is measured.^26,28^ The adsorption amount of C_60_ on the lipid membranes has not been reported in previous studies. Furthermore, the degree of membrane damage caused by C_60_ possibly be quantified using QCM-D and specially designed model membranes at the first time. Then we can know fullerene NPs cause more or less serious membrane damage compared with another type of NPs.

The surface charge of NPs is an important factor in the NP-induced membrane damage because charged NPs usually interact with the oppositely charged groups in the membrane.^29^ The role of electrostatic force in C_60_–membrane interaction is worth investigations. Fluid-phase cell membranes are essential to keep the normal cellular activities.^30^ Nanoparticles exposure has been reported either to cause membrane gelation,^24,31^ or increase membrane fluidity.^32^ C_60_ may cause unique effects on membrane fluidity, which needs to be evaluated in this study. Therefore, this study aims to evaluate the influences of C_60_ on membrane integrity, morphology and fluidity; and aim to build up a method to quantify the degree of membrane damage. The results will provide better understanding to the fullerene–membrane interaction and the related cytotoxic mechanisms.

## 2. Materials and methods

### 2.1. Materials

Fullerene C_60_ (purity > 98%) was purchased from Sigma-Aldrich Co. LLC (St. Louis, MO, USA). 1,2-Dioleoyl-*sn*-glycero-3-phosphocholine (DOPC), positively charged 1,2-dipalmitoyl-3-trimethylammonium-propane (chloride salt) (16:0 TAP), negatively charged 1,2-dioleoyl-*sn*-glycero-3-[phosphor-*rac*-(1-glycerol)] (sodium salt) (DOPG), and the fluorescence labeled lipid 1,2-dipalmitoyl-*sn*-glycero-3-phosphoethanolamine-*N*-(lissamine rhodamine B sulfonyl) (RhB-PE) were purchased from Avanti Polar Lipids (Alabaster, AL, USA). The molecular structures of DOPC, 16:0 TAP and DOPG are presented in ESI.† The fluorescent probe 6-dodecanoyl-2-dimethylaminonaphthalene (laurdan) was purchased from Molecular Probes (Eugene, OR, USA) to evaluate the membrane phase.

### 2.2. Preparation of C_60_ suspension and characterization

**Fig. 1:**
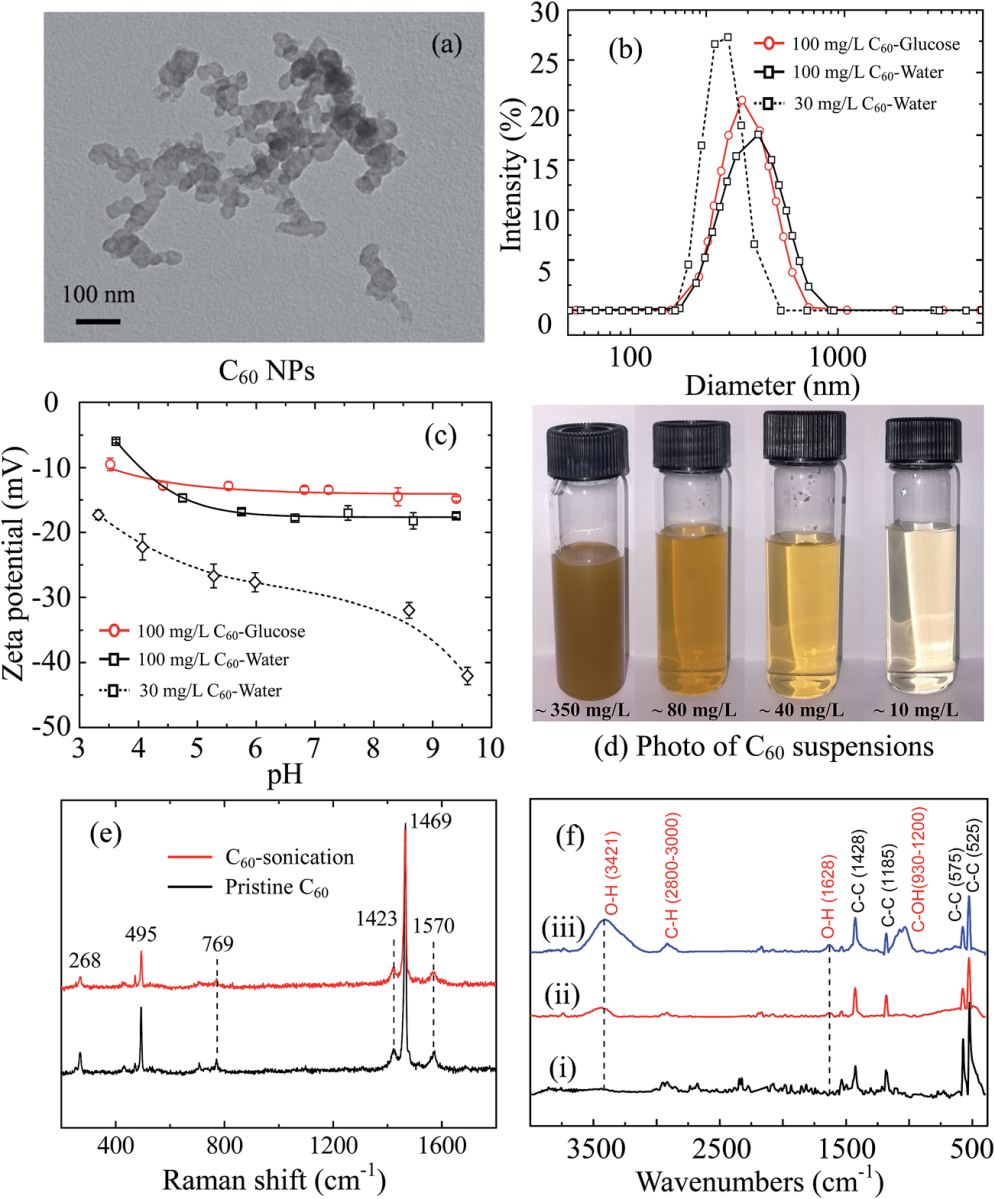
Characterization of C_60_ NPs. (a) TEM imaging. (b) Size distribution of 30 mg L^−1^, 100 mg L^−1^ C_60_ suspension in DI water and 100 mg L^−1^ C_60_ suspension in 0.1 M glucose. (c) Zeta potentials of 30 mg L^−1^, 100 mg L^−1^ C_60_ suspensions in DI water and 0.1 M glucose at pH 3–10. (d) Photos for the supernatant obtained from the C_60_ suspension after 4 hour sonication. (e) Raman spectrum of the pristine C_60_ powder and sonicated C_60_ sample (4 hour sonication). (f) The FTIR spectra of pristine C_60_ powder as purchase (i), C_60_ sonicated for 4 hours (ii), and C_60_ sonicated for 10 hours (iii).

Fullerene is extremely hydrophobic and insoluble. Fullerene stock suspension (100 mg L^−1^) was prepared by weighting 10 mg C_60_ powder, and bath sonication (40 kHz) in 100 mL deionized (DI) water or in 0.1 M glucose for 4 hours at room temperature. The stock suspensions were adjusted to pH 6.5 and stored at 4 °C, which were used to interact with GUVs and were sonicated again for 30 min before experiments. The suspensions used in QCM-D measurements were obtained from the stock fullerene suspension in DI water. The supernatant was collected from the stock suspension after standing 24 hours in darkness, which was approximately 60–70 mg L^−1^ quantified by a UV-visible spectrophotometer. The supernatant was diluted by DI water to 30 mg L^−1^, and was sonicated for 30 min to obtain a yellow suspension (Fig. 1d). It was sonicated again for 30 min before QCM-D experiments.

The morphology of C_60_ aggregates was imaged by a transmission electron microscopy (TEM, JEM-1011, JEOL Ltd., Japan). To prepare TEM specimens, a tiny drop of C_60_ suspension was put on a copper mesh grid, and dried under an infrared lamp. The imaging was performed at an electron emission of 100 kV. The hydrodynamic diameter (*d*_H_) and zeta potentials of C_60_ NPs were measured by a Malvern Zetasizer (Nano ZS90, Malvern Instruments Ltd., UK). The Raman spectrum of pristine and sonicated C_60_ (4 hours) was obtained at the excitation wavelength of 532 nm *via* a Raman spectrometer (inVia Reflex, Renishaw plc, UK). Fourier transform infrared (FTIR) spectra were obtained using a FTIR spectroscopy (ALPHA-T, Bruker Co., Germany) by collecting more than 50 scans at a resolution of 4 cm^−1^. Three C_60_ samples were prepared for IR scans: C_60_ powder as purchased, C_60_ stock suspension in DI water sonicated for 4 hours and for 10 hours and then freeze dried. The collected C_60_ samples were mixed with spectrum pure potassium bromide and pulverized, then squashed seconds into a chip under 10 MPa pressure for infrared transmission testing.

### 2.3. Preparation of GUVs

Gaint unilamellar vesicles were prepared by the gentle hydration method using DOPC and charged lipids.^33^ 10% (w/w) 16:0 TAP (positively charged) or DOPG (negatively charged) was added into DOPC solution in 2 : 1 (v/v) chloroform : methanol to obtain positively/negatively charged GUVs. To prepare RhB-labeled GUVs, 0.1% (w/w) of RhB-PE was added to the lipid solution. The lipid solution (50 μL, 10 mg mL^−1^) was dried under N_2_ gas in a 4 mL glass vial to form a film inside the vial. Then the vial was kept in a desiccator in vacuum for 2 hours to remove the residual organic solvent. Finally the vial was filled with 0.1 M sucrose and incubated at 40 °C for 24 hours to obtain the stock solution of GUVs.

### 2.4. Fullerene exposure to GUVs and the microscopic observation

Fullerene suspension (100 mg L^−1^) in 0.1 M glucose was prepared before experiments. GUV stock solution of 10 μL was mixed with 390 μL C_60_ suspension in a Petri dish with a glass bottom (30 mm diameter, 0.15 mm thickness). The GUV concentration in the Petri dish was 100 mg L^−1^. Morphologies of GUVs were recorded from 30 min to 24 h under bright field by an inverted microscope with a 40× objective lens. During the exposure experiment, 0.1 M sucrose inside GUVs and 0.1 M glucose outside GUVs had different refractive indices, which made GUVs visible under bright field. Moreover, RhB-labeled GUVs were applied to observe the broken GUVs and lipid fragments caused by C_60_ exposure. Fluorescent images were taken by a confocal laser microscopy (LSM 700, Zeiss, Germany), and RhB-PE probes were excited by 555 nm wavelength laser.

### 2.5. QCM-D measurement to evaluate C_60_–membrane interaction

The C_60_ adhesion on model membrane was monitored using a QCM-D at 25 °C (E4, Q-Sense, Sweden). Before experiments, supported lipid bilayer (SLB) or a layer of small unilamellar vesicles (SUVs) was prepared on QCM sensor as the model membrane.^34^ SUVs (50 nm) were produced though extruding method,^35^ which was introduced in detail in the ESI.† DI water was first injected into the measurement chamber mounted with SiO_2_ crystal sensor (5 MHz, work area: 0.78 cm^2^) to create a baseline, and then Tris/NaCl buffer (10 mM Tris, 150 mM NaCl, pH 7.0 ± 0.2) was injected as the background solution. After the baseline of Tris/NaCl buffer became stable, 100 mg L^−1^ SUV suspension was injected into the chamber, finally Tris/NaCl buffer was injected again to remove the residual vesicles in the flow module, and SLB was formed on the silica-coated sensor. The flow rate was 70 μL min^−1^ during the whole process. To prepare SUV-coated sensor, SUVs of 100 nm in diameter were prepared through the same extruding method. Similar to the process of SLB preparation, DI water, Tris/NaCl buffer, 100 mg L^−1^ SUV suspension and Tris/NaCl buffer were successively injected into the chamber, but the chamber was mounted with a gold crystal sensor (5 MHz, work area: 0.78 cm^2^) and the flow rate was 100 μL min^−1^. A layer of SUVs was formed on the gold sensor.

After the SLB or SUV layer was prepared on the crystal sensor, DI water was injected again to obtain a baseline, and then 30 mg L^−1^ C_60_ suspension in DI water (pH 6.5) was injected into the chamber at the rate of 100 μL min^−1^ for 1 h. Resonant frequency (*f*) and dissipation (*D*) responses of crystal sensors at *n*th overtones (*n* = 3, 5, 7, 9, 11) were monitored throughout the experiment, and frequency and dissipation shifts (Δ*f* and Δ*D*) at the 3rd overtone were used for the data processing.

To calculate the mass of C_60_ NPs deposited on model membranes, an appropriate model was needed. For thin and rigid films like SLBs, the Δ*D* is nearly zero, and the Δ*f* is proportional to the mass change on the sensor surface, as described by the Sauerbrey equation:^36,37^
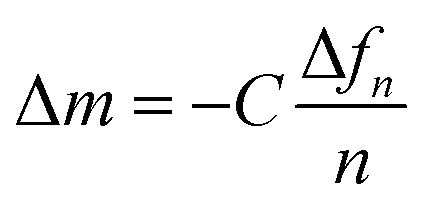


Δ*m* is the adsorbed mass per unit surface, *C* is the mass sensitivity constant (17.7 ng Hz^−1^ cm^−2^ when the quartz crystal frequency is 5 MHz), *n* is the overtone number and Δ*f*_*n*_ is the frequency shift at the *n*th overtone. For the viscoelastic and soft layer deposited on the crystal sensor, the Sauerbrey equation underestimates the mass change. Instead the Voigt model was applied to quantify the mass change on a viscoelastic layer,^38–40^ which was fitted by the QTools software (Q-Sense, Sweden). The Voigt model is adapted to analyze the mass, viscosity, thickness of viscoelastic layers.^41,42^ Δ*f* and Δ*D* values were input into model, and fluid density, viscosity and the density of the deposited layer were needed as fitting constants. The fluid density and viscosity were 1.00 × 10^3^ kg m^−3^ and 1.00 × 10^−3^ kg (m s)^−1^. The density of the deposited lipid layer was 1.10 × 10^3^ kg m^−3^,^43^ and the density of C_60_ layer was 1.05 × 10^3^ kg m^−3^.^40^ More details of QCM-D experiments and data processing are provided in the ESI.†

### 2.6. Membrane phase quantification after C_60_ exposure

In order to study the influence of C_60_ NPs on the membrane fluidity, 0.4% (w/w) of laurdan probe was added into the lipid bilayers during the GUV preparation.^24^ Laurdan-labeled GUVs of 50 mg L^−1^ were then exposed to 100 mg L^−1^ C_60_ suspension. The fluorescence spectra of GUVs were measured by a fluorescence spectrophotometer (F7000, HITACHI, Japan) at 1 h and 24 h, respectively. The sample was excited at 380 nm, and the emission spectrum was collected from 400 nm to 600 nm. Generalized polarization (GP) was used to quantify the phase of phospholipid membranes,^24,44,45^ which was defined as:
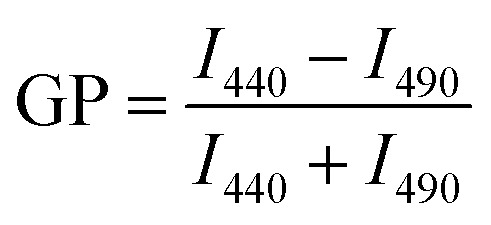


*I*_440_ and *I*_490_ are the emission intensities at 440 nm and 490 nm, respectively. GP values range from −1 to 1. The membranes are in gel phase when GP > 0.55, and in fluid phase when GP < −0.05. GP value between the two ranges (−0.05 < GP < 0.55) indicates the intermediate phase.^46^

## 3. Results and discussion

### 3.1. Characteristics of C_60_ powder and suspension

Fullerene C_60_ forms clusters of tens of nanometers revealed by TEM images (Fig. 1a). The C_60_ clusters are stable crystalline form with reported diameters of 25–500 nm in water,^6^ which is much larger than C_60_ molecule (0.71 nm in diameter).^47,48^ The measured average *d*_H_ of 100 mg L^−1^ C_60_ stock suspension is 453.5 ± 5.1 nm in 0.1 M glucose and 409.6 ± 13.6 nm in DI water with broad size distribution (Fig. 1b). The two C_60_ stock suspensions are negatively charged in the whole measured pH range (pH 3–10) (Fig. 1c). The diluted C_60_ supernatant in water (30 mg L^−1^) has smaller average *d*_H_ (256.9 ± 1.4 nm) with narrower size distribution, as well as more negative zeta potentials, suggesting a better dispersion.

Although pristine C_60_ molecules with perfect structure do not contain functional groups, they can extend to water *via* sonication or long-term mixing in water for a period of days to weeks.^49,50^ C_60_ molecules are strong electron accepters, and interact with the potential electron donors like water molecules to become negatively charged.^51,52^ The adsorption of water molecules on pristine C_60_ clusters forms hydrated layer and improve its solubility.^52,53^ The diluted C_60_ supernatant (30 mg L^−1^) shows negligible sedimentation in initial 2 hours and stable hydrodynamic diameter after 30 min sonication (ESI Fig. S1c and d†), suggesting C_60_ aggregates can be stably suspended in supernatant.

Furthermore, the Raman spectra of pristine C_60_ powder and sonicated C_60_ are presented in Fig. 1e. Both pristine and sonicated C_60_ show Raman-active A_g_ (495 and 1469 cm^−1^) and H_g_ vibrational modes (268, 769, 1423 and 1570 cm^−1^),^54,55^ indicating no distinct structural defects. The FTIR spectra (Fig. 1f) show sharp peaks at 525, 575, 1185 and 1428 cm^−1^, which derives from the fundamental IR-active vibrational modes of C_60_ skeleton with icosahedral symmetry.^56,57^ After 4 hour sonication, new distinguishable absorb peaks emerge at 3421 and 1628 cm^−1^ corresponding to O–H groups,^58^ which enhances with longer sonication time (10 hours). Another band appears from 930 to 1200 cm^−1^ after 10 hour sonication, corresponding to C–O groups with various chemical environments.^58^ It indicates that 10 hour sonication may induce more serious structural changes compared to 4 hour sonication. A slight absorbance increase is detected at the range of 2800–3000 cm^−1^, which is caused by the formation of C–H bonds.^59^ Therefore, sonication increases the hydroxyl groups on C_60_ surface, which makes C_60_ more hydrophilic and contributes to the dispersion of C_60_. The form of surface hydroxyl is attributed to the hydrogen radicals that generated from water by sonication.^57^ Compare to 10 hour sonication, 4 hour sonication effectively stabilizes C_60_ suspension and does not cause serious C_60_ structure damage (confirmed by Raman spectrum), which is more suitable for pristine C_60_ study.

### 3.2. Effects of C_60_ on GUVs revealed by microscopic imaging

**Fig. 2:**
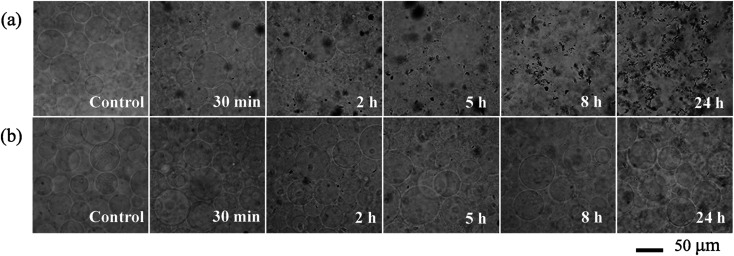
Bright field images of GUVs^+^ (a) and GUVs^−^ (b) after exposure to 100 mg L^−1^ C_60_ NPs in 0.1 M glucose.

**Fig. 3:**
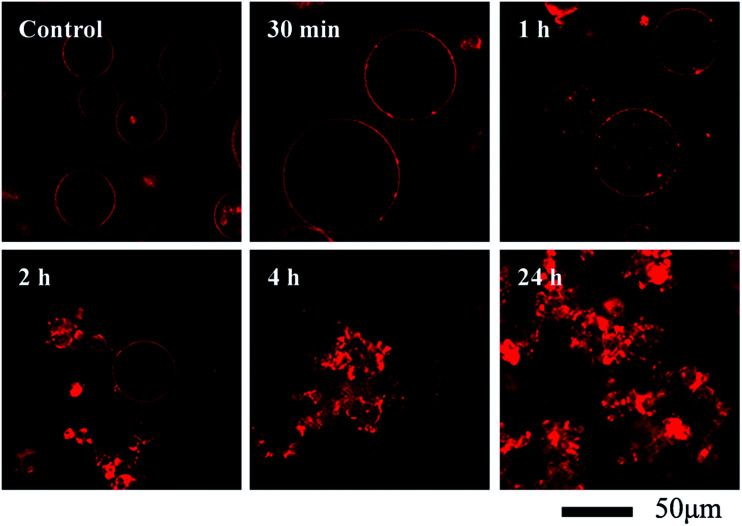
Morphological changes of RhB-labeled GUVs^+^ after exposure to 100 mg L^−1^ C_60_ NPs in 0.1 M glucose.

Giant unilamellar vesicles are exposed to C_60_ suspension in 0.1 M glucose at pH 6.5, and their morphology is imaged under bright field (Fig. 2). Both GUVs^+^ and GUVs^−^ are 20–100 μm spheres under microscopy. However, there is big difference between GUVs^+^ and GUVs^−^ after C_60_ exposure. C_60_ NPs form obvious aggregates and adhere on the GUVs^+^ at 2 hour (Fig. 2a). Serious membrane disruption appears at 8 hour, and few GUVs exist when it comes to 24 hour. GUVs are broken into lipid fragments and aggregate with C_60_ NPs, which appears as the black spots on the images. The broken GUVs are not visible under bright field due to the leakage of the enclosed sucrose. Therefore RhB-labeled GUVs are used to show the morphology of disrupted GUVs. The formation of lipid agglomerates from disrupted GUVs is revealed by the fluorescence imaging (Fig. 3), indicating that C_60_ adhesion causes lipid fragments to attach to each other. However, GUVs^−^ are not disrupted by C_60_ up to 24 hours, and no C_60_ aggregates or lipid fragments are found in the images (Fig. 2b and ESI Fig. S2†).

Cell membranes contain both positively and negatively charged domains, the positively charged domains are relatively scarcer than negatively charged ones.^29^ In the GUV exposure experiment, the zeta potentials of 100 mg L^−1^ C_60_ are −17.5 mV in DI water and −13.7 mV in glucose at pH 6.5 (Fig. 1c). Negatively charged C_60_ NPs only adhere on and damage the membranes containing positively charged groups. The positively charged sites on the membrane seem to be necessary for C_60_ NPs approaching to the membrane and for the consequent membrane damage. It indicates that negatively charged NPs can approach to the cationic sites on the plasma membrane through nonspecific binding, which can explain the strong and nonspecific interaction of anionic NPs with the plasma membrane and their subsequent endocytosis reported in previous studies.^60,61^

### 3.3. C_60_ adhesion on model membrane and induced membrane disruption monitored by QCM-D

**Fig. 4:**
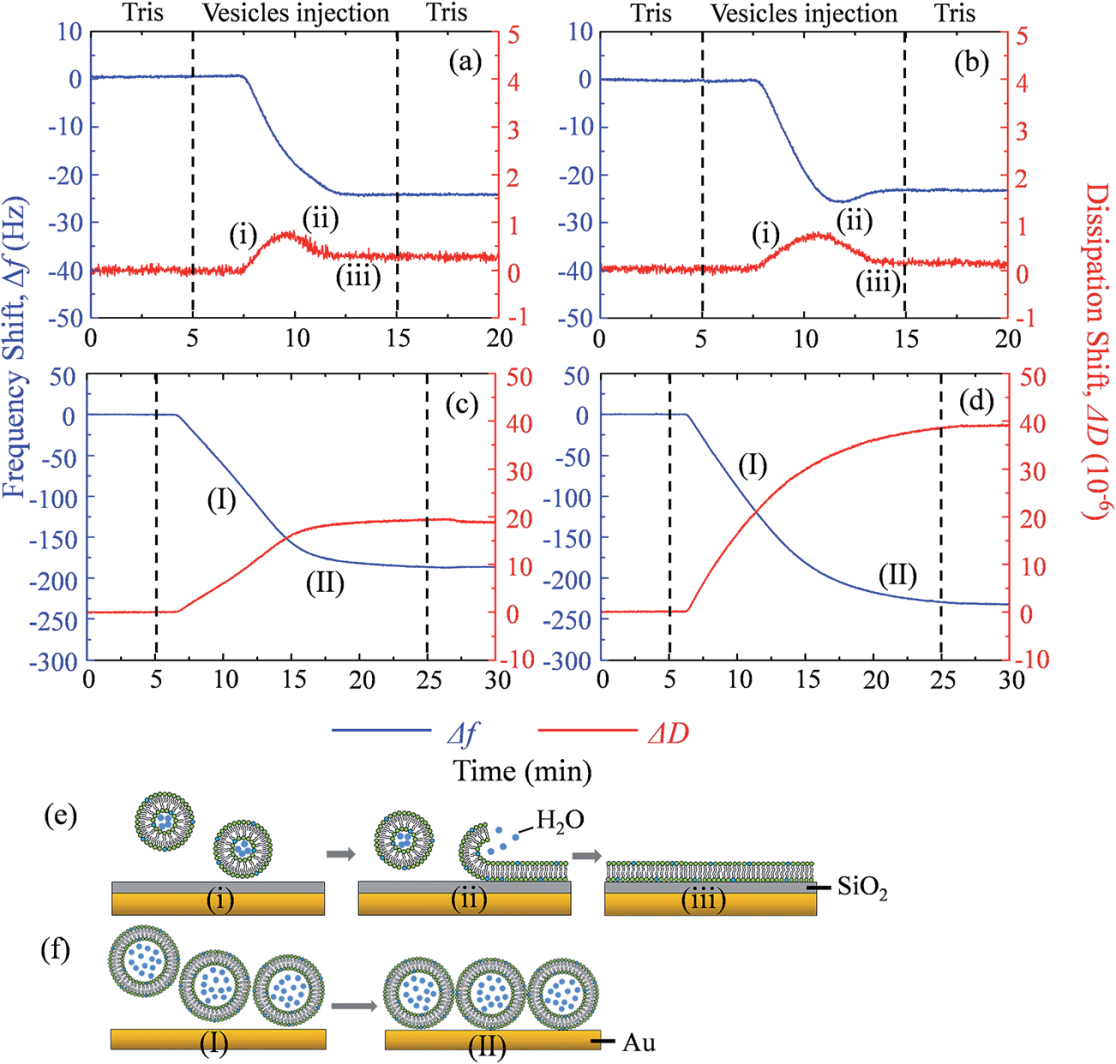
Frequency shifts (Δ*f*, blue) and dissipation shifts (Δ*D*, red) during the formation of SLB^+^ (a), SLB^−^ (b), SUV^+^ layer (c) and SUV^−^ layer (d) at pH 7.0. The formation of SLB on a silica sensor (e) and the formation of a SUV layer on a gold sensor (f) are illustrated.

**Fig. 5:**
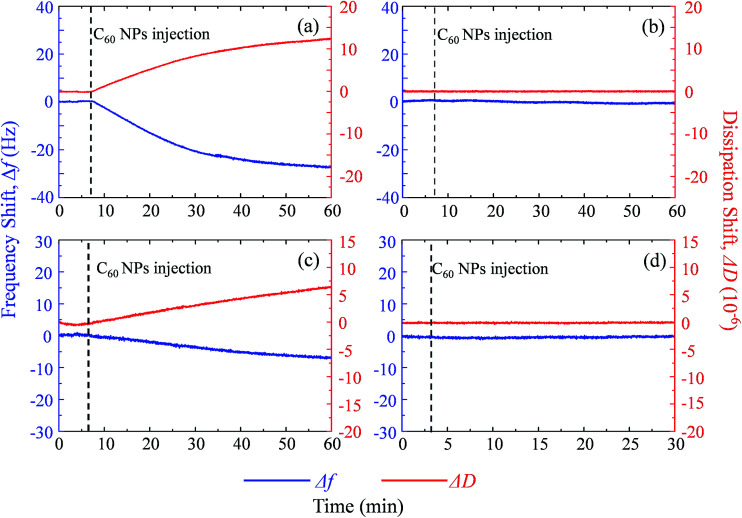
The frequency shift (Δ*f*, blue) and dissipation shift (Δ*D*, red) during C_60_ deposition on SLB^+^ (a), SLB^−^ (b), SUV^+^ layer (c) and SUV^−^ layer (d) at pH 6.5.

Supported lipid bilayer on SiO_2_ sensor is designed to show the mass of adhered C_60_ on membrane, while a SUV layer on Au sensor is designed to reveal the combined mass change of C_60_ adhesion and vesicle disruption. To prepare SLBs on QCM-D sensor, SUVs are injected into QCM chamber and then disrupt quickly to form a continuous SLB on the SiO_2_ sensor. Δ*f* and Δ*D* provide the information of mass change and viscoelastic properties of the deposited layer, respectively.^41^ During SLB formation process, Δ*f* decreases because of the vesicle deposition, and then becomes stable after SLB formation (Fig. 4a and b). It indicates that no more phospholipids adsorb on the sensor after the sensor has been fully covered by the lipid bilayer. The vesicle disruption induces a peak on Δ*D* curve because of the different viscoelasticity between the adhered intact vesicles and the consequently formed phospholipid bilayer. Δ*D* just slightly increases after SLB formation and Δ*D*/Δ*f* is less than 1 × 10^−8^ Hz^−1^ (Table S1†), indicating that SLB is a rigid film.^34^ When 30 mg L^−1^ C_60_ suspension is injected to the SLB^+^-coated sensor, Δ*f* decreases by 27.5 Hz and Δ*D* increases by 12.5 × 10^−6^ after one hour (Fig. 5a), indicating the fast deposition of C_60_ NPs on the SLB^+^. To prepare the layer of SUVs, intact SUVs deposit on the Au sensor, leading to the decrease of Δ*f* and the increase of Δ*D* (Fig. 4c and d). Different from SLB, the layer of SUVs is a viscoelastic film (Table S1,† Δ*D*/Δ*f* > 1 × 10^−8^ Hz^−1^).^34^ After C_60_ injection, a decrease of Δ*f* and an increase of Δ*D* are also observed on SUV^+^-coated sensor (Fig. 5c), indicating the deposition of C_60_ NPs on SUVs^+^.

Fullerene suspension is also injected to uncoated SiO_2_ and Au sensor to compare with the membrane-coated sensor (Fig. S3†). No deposition is observed on the uncoated sensor, which confirms that changes of Δ*f* and Δ*D* on SLBs/SUVs are due to the interaction between C_60_ NPs and the membrane. Based on the relatively high Δ*D*/Δ*f* values of C_60_ deposition on SLB and SUV layer (Table S1†), the deposited C_60_ NPs form a viscoelastic film on both SLBs^+^ and SUVs^+^. Therefore the Voigt model is employed to calculate the mass of adhered C_60_ NPs on the SLB/SUV-coated crystal sensor.^41,42^

**Fig. 6:**
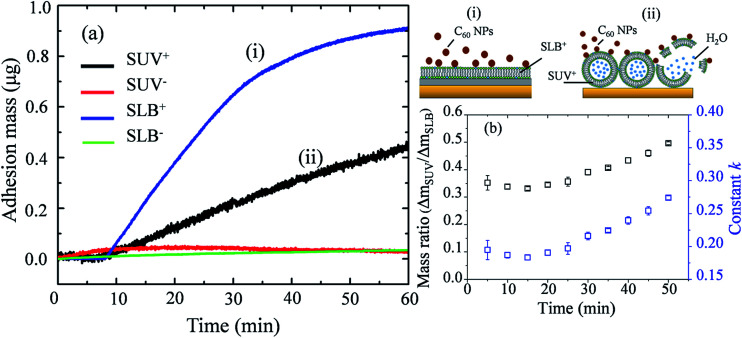
(a) Mass changes on SUV-coated (Δ*m*_SUV_) and SLB-coated (Δ*m*_SLB_) crystal sensors when C_60_ NPs are injected into the measurement chamber of QCM-D. (i) The illustration for the deposition of C_60_ NPs on SLB^+^; (ii) the illustration for the deposition of C_60_ NPs on SUV^+^ layer and the disruption of SUVs. (b) The calculated Δ*m*_SUV_/Δ*m*_SLB_ and constant *k* for C_60_ NPs.

Mass changes on the SLB- or SUV-coated crystal sensors during C_60_ injection are presented in Fig. 6a. After 1 hour injection, the masses of adhered C_60_ on SLBs^+^ (Δ*m*_SLB_) and on SUVs^+^ (Δ*m*_SUV_) are calculated to be 0.9226 μg and 0.4466 μg on sensor, respectively. The mass increase on SLB^+^ is remarkably higher than on SUV^+^ layer. The surface area of SUV layer (*A*_SUV_) is calculated to be 1.42 cm^2^ on QCM sensor (Fig. S4†), which is larger than the surface area of SLB (*A*_SLB_ = 0.78 cm^2^). Hence the ratio of adhesion mass on SUV^+^ to SLB^+^ (Δ*m*_SUV_/Δ*m*_SLB_) should be proportional to the ratio of surface area (*A*_SUV_/*A*_SLB_ = 1.81).
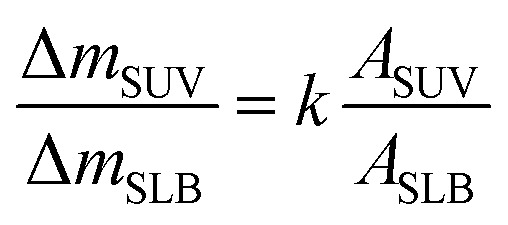


If C_60_ deposition on sensor only cause particle adhesion on the two types of model membranes, the constant *k* should be 1 at a given injection time. However, Δ*m*_SUV_/Δ*m*_SLB_ of C_60_ is between 0.33 and 0.50 (Fig. 6b), and the constant *k* is between 0.18 and 0.28 (much less than 1). It indicates that partial SUVs are disrupted by C_60_, therefore the removal of lipid fragments and the release of the enclosed fluid from SUVs cause mass loss. Imaging on GUV morphology confirms that C_60_ NPs disrupt positively charged vesicles (Fig. 2 and 3), however a quantitative way is required to evaluate how serious the membrane damage is and to compare the biological risks between different NPs. The constant *k* can be the used to quantify the membrane disruption, and the smaller *k* value means more serious membrane disruption. We also calculated Δ*m*_SUV_/Δ*m*_SLB_ and constant *k* values of CdTe quantum dots (QDs) using the QCM monitored mass data in a previous study.^62^ The Δ*m*_SUV_/Δ*m*_SLB_ of amino-coated QDs is between 1.44 and 2.21, and the constant *k* is between 0.79 and 1.22 (Fig. S5a and b†). Δ*m*_SUV_/Δ*m*_SLB_ of carboxyl-coated QDs is between 1.16 and 1.34, and the constant *k* is between 0.64 and 0.74 (Fig. S5c and d†). Therefore C_60_ cause more serious membrane disruption than CdTe QDs. However, the mass loss induced by SUV disruption does not exceed the mass increase caused by C_60_ adhesion, hence only a small part of SUVs are disrupted and Δ*f* is governed by C_60_ adhesion.

On SLB^−^ and SUV^−^ layer, C_60_ injection does not cause obvious Δ*f* and Δ*D* change (Fig. 5b and d), and results in almost zero mass increase in Fig. 6. It suggests that C_60_ NPs do not adhere to the membrane containing negatively and neutrally charged lipids. C_60_ NPs only adhere on SLB^+^ or SUV^+^ layer containing positively charged lipids, which is consistent with the microscopic observation using GUVs.

Experiments using GUV, SUV and SLB model membranes have confirmed that negatively charged C_60_ NPs only adhere on and disrupt membranes containing positively charged lipids, indicating the importance of the electrostatic force in the interaction between C_60_ NPs and lipid membranes. Most biological membranes are negatively charged. However, there are still a small amount of cationic sites on the cell membranes,^29,47^ hence negatively charged NPs can bind to the cationic sites and cause the subsequent cell membrane damage. Studies on the interaction of lipid membranes with other carbon nanomaterials (single-walled and multi-walled carbon nanotubes, graphene oxides) also demonstrate the role of the electrostatic force.^26,63,64^ Therefore, the electrostatic force is a crucial mechanism in the interaction of carbon nanomaterials with lipid membranes.

### 3.4. Influence of C_60_ NPs on membrane fluidity

Fluid phase cell membrane is essential to support membrane proteins and to maintain normal molecular transport into and out of cells.^10^ Laurdan is a fluorescent probe which is sensitive to the polarity of the surrounding environment. The fluorescent spectra of laurdan emission and GP values are presented in Fig. S6.† In gel-phase membrane few water molecules exist inside phospholipid bilayers, therefore the maximum of laurdan emission is about 440 nm.^45^ In fluid-phase membrane, more water molecules exist between phospholipid molecules and cause the dipolar relaxation of laurdan. The maximum laurdan emission shifts to 490 nm.^45^ Negatively charged NPs usually cause membrane phase gelation because they attract the –N^+^ terminus of the phospholipid head groups and increase the angle of P^−^–N^+^ electric dipole.^65^ However, the prepared GUVs^+^ and GUVs^−^ are both in fluid phase (GP < −0.05), and the exposure of 100 mg L^−1^ C_60_ NPs does not change the GP values significantly. Such particles inducing membrane phase gelation have been reported for carboxyl-modified polystyrene latex and silica NPs (∼20 nm in diameter).^24,65^ In contrast to those NPs, C_60_ molecules reside on the border between molecular level chemicals and nanomaterials (Fig. 1c). They penetrate into the interior of membranes and then remain encased in the hydrophobic lipid tails revealed by both molecular dynamic simulations and experimental confirmation.^13,14,66,67^ C_60_ forms stable aggregates in water. Transferring one C_60_ from the aggregate into bulk water is highly unfavorable, but removing one C_60_ from a large aggregate and placing it into the lipid bilayer interior is favorable.^12^ Large C_60_ aggregates adsorb on the lipid membranes, and partially disaggregate into small aggregates or single C_60_ molecules which can penetrate into lipid bilayers.^68^ This special process differs to the adhesion of common charged NPs, possibly prevents the gap narrowing between phospholipid chains and membrane gelation. In summary, the particle attachment induced membrane gelation is not a concern for the cytotoxicity of C_60_ NPs.

## 4. Conclusions

In summary, fullerene C_60_ disrupts the positively charged membranes, but not the negatively charged membranes, suggesting the interaction between C_60_ and the cationic moieties in the membrane. The degree of the induced membrane disruption is evaluated by the mass increase on SLB and on SUV layer. SUV layer and SLB have same lipid components, however the value of Δ*m*_SUV_/Δ*m*_SLB_ after C_60_ deposition is much smaller than the value of *A*_SUV_/*A*_SLB_ (constant *k*: 0.18–0.28). It indicates some of SUVs have been disrupted by C_60_. The smaller constant *k* suggests more serious membrane disruption. In this way, the membrane disruption induced by C_60_ can be quantified and compared with other types of NPs. It is important to know which type of NPs causes more serious membrane disruption, which is helpful for the biological risk evaluation and the safe application of nanomaterials.

## Conflicts of interest

There are no conflicts to declare.

## Supplementary Material

RA-008-C7RA13690K-s001
