# Supplementary material for: Quantification of C60-induced membrane disruption using a quartz crystal microbalance
Source: RSC Adv. 2018 Mar 9;8(18):9841–9. doi: 10.1039/c7ra13690k (PMC9078712; doi:10.1039/c7ra13690k)
Supplement: RA-008-C7RA13690K-s001 [file RA-008-C7RA13690K-s001.pdf]

Electronic Supplementary Material (ESI) for RSC Advances.
This journal is © The Royal Society of Chemistry 2018

Supplementary information:

Quantification of C_{60}-induced Membrane Disruption Using Quartz Crystal Microbalance

Yuxuan Zeng[a], Qi Wang[a], Qiu Zhang[b], Wei Jiang[a,*]

[a]Environment Research Institute, Shandong University, Jinan, 250100, China

[b]School of Chemistry and Chemical Engineering, Shandong University, Jinan, 250100, China

*Corresponding author. Tel.: 86-531-88366072; Fax: 86-531-88361990; E-mail address: jiangw@sdu.edu.cn (Wei Jiang).

The molecular structures of phospholipids and probes:

1,2-dioleoyl-sn-glycero-3-phosphocholine (DOPC):

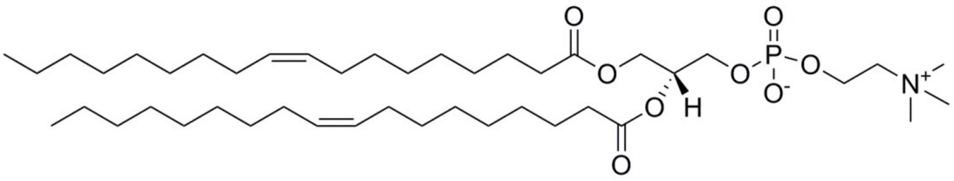

1,2-dioleoyl-sn-glycero-3-[phosphor-rac-(1-glycerol)] (sodium salt) (DOPG):

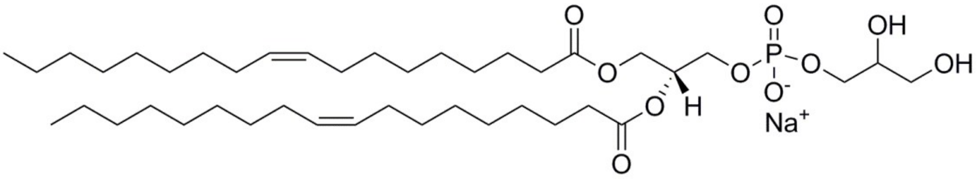

1,2-dipalmitoyl-3-trimethylammonium-propane (chloride salt) (16:0 TAP):

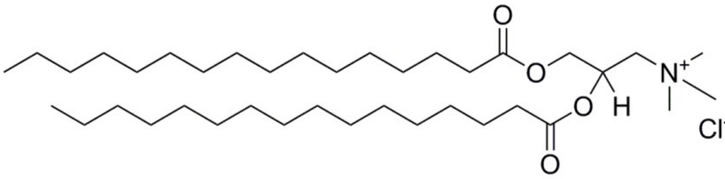

1,2-dipalmitoyl-sn-glycero-3-phosphoethanolamine-N-(lissamine rhodamine B sulfonyl) (RhB-PE):

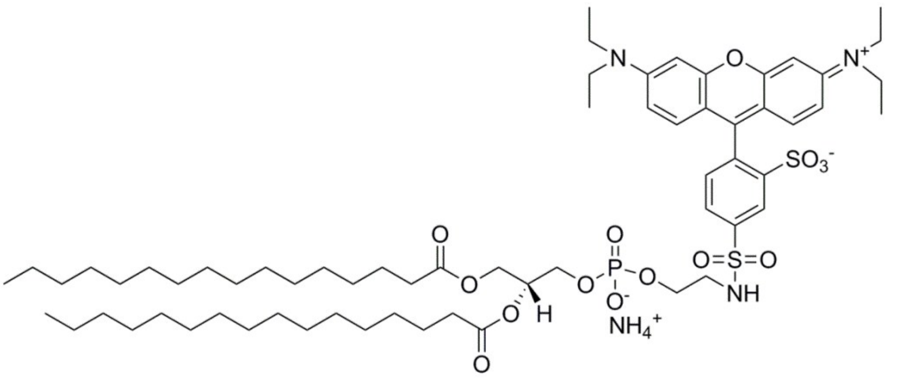

6-dodecanoyl-2-dimethylaminonaphthalene (Laurdan):

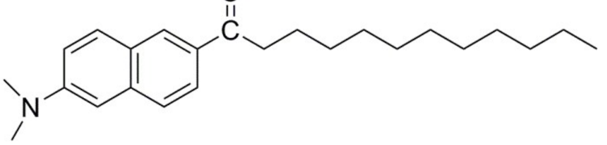

Details of QCM-D Experiments

Solution preparation

Tris/NaCl buffer (10 *mM* Tris, 150 *mM* NaCl) was prepared in deionized (DI) water and adjusted to pH 7.0 ± 0.2. The buffer was filtered by a 0.2 μm filter (Satorius, Germany) before use. SDS solution (2%, w/w) was used to clean the flow modules of QCM-D and the silica-coated crystal sensors.

Preparation for the stock suspension of small unilamellar vesicles (SUVs)

Suspensions of vesicles with a diameter of 100 and 50 nm were used for the formation of SUVs and SLBs, respectively.[1] DOPC solution (0.2 mL) was mixed with another 0.2 mL 16:0 TAP or DOPG solution in a 15 mL glass vial to make positively-charged or negatively-charged vesicles. The mixture of lipids was dried under nitrogen gas blow and a film of lipids was formed inside the vial. The vial was placed in a vacuum desiccator for 6 h to remove the residual organic solvent. The lipids in the vial were hydrated in 1 mL DI water for 30 min at 40 °C to obtain the lipid suspension with a concentration of 4 mg/mL. To obtain the stock suspension of SUVs, a mini-extruder (Avanti Polar Lipids, USA) was used to treat the lipids in the vial. The lipid suspension was extruded forth and back through a filtration membrane with an aperture size of 100 or 50 nm for more than 31 times[1]. The SUV suspension of 4 mg/mL was diluted by Tris/NaCl buffer to the concentration of 0.1 mg/mL before the injection into the QCM-D flow modules.

Cleaning of the instrument and crystal sensors

Flow modules of QCM-D were rinsed with SDS solution and a large volume of DI water, and were dried in nitrogen gas. For silica-coated crystal sensors, the sensors were first treated by UV/ozone cleaner for 10-20 min, and immersed in the SDS solution for 30 min at room temperature. Next the sensors were rinsed in DI water and dried in nitrogen gas. Finally sensors were treated in the UV/ozone cleaner again for 10-20 min. To clean the gold crystal sensors, DI water, 25% ammonia and 30% hydrogen peroxide were mixed at the ratio of 5:1:1, and heated to 75 °C. The Au

sensors were treated by UV/ozone for 10-20 min, then were placed in the above-mentioned solution for 5 min, and were rinsed in DI water. Finally the sensors were dried in nitrogen gas and treated by UV/ozone for another 10-20 min.

QTools modeling

The Voigt model was used to calculate the mass of C_{60} adsorbed onto model membranes.[2] Since there were two layers on the crystal sensor: the membrane layer and the C_{60} NP layer, the viscoelastic models for two layers were used. For C_{60} adsorption on SLBs, the first layer was a rigid film. The experimental data were offset at a data point before the C_{60} NP suspension was added. Then the Voigt model was used to fit the data of C_{60} adsorption step to get the value of mass. For C_{60} adsorption on the SUV layer, the first layer was a viscoelastic film. The Voigt model was used to fit the values of the SUV layer and C_{60} NP layer one by one. By fitting the step of SUV layer formation, the viscosity, shear module and mass change of the SUV layer were obtained. Then the parameters obtained were used as constants to fit the data of C_{60} adsorption step, and the value of C_{60} mass adsorbed onto the SUV membrane was calculated.

Table. S1. The rigidity of deposited films on the QCM crystal sensors evaluated by *ΔD/Δf*

Film on QCM sensor	*ΔD/Δf* (Hz^{-1})	Film rigidity
$SLBs^{+}$	8.24×10^{-9}	< 1×10^{-8}, rigid film
$SLBs^{-}$	9.93×10^{-9}	
$SUVs^{+}$	1.69×10^{-7}	> 1×10^{-8}, elastic film
$SUVs^{-}$	1.01×10^{-7}	
C_{60} on $SLBs^{+}$	4.50×10^{-7}	> 1×10^{-8}, elastic film
C_{60} on $SUVs^{+}$	9.44×10^{-7}	

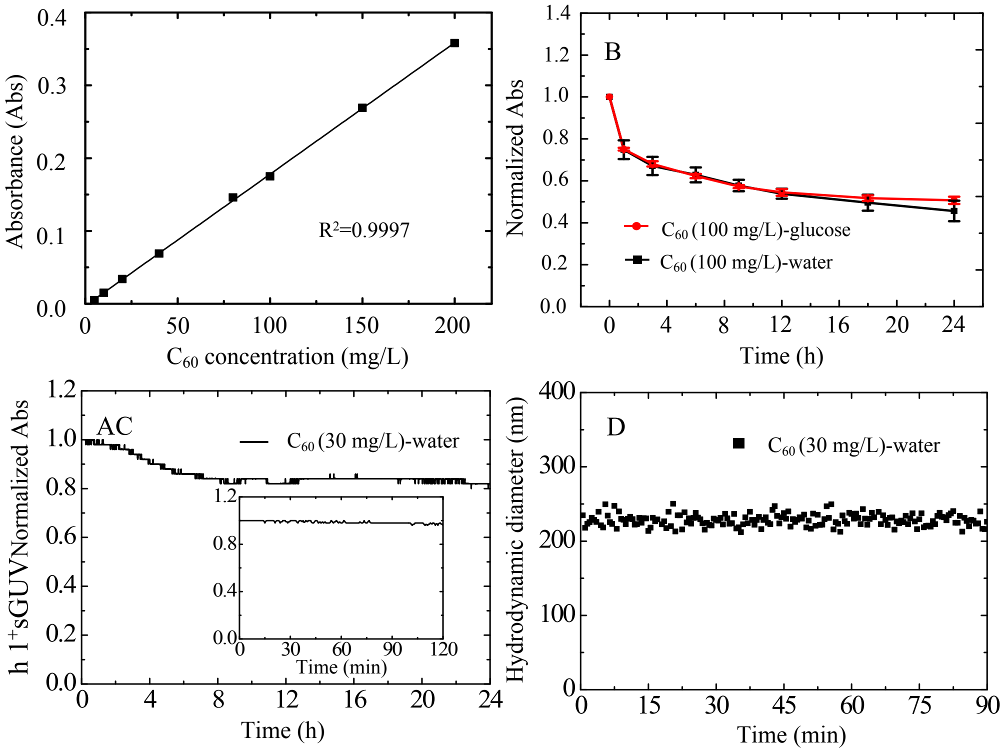

Fig. S1. (A) The standard curve of C_{60} suspension with concentration from 5 to 200 mg/L. The absorbance of C_{60} suspension was measured at 800 nm after additional 30-minute sonication. (B) The curves of normalized absorbance of 100 mg/L C_{60} stock suspensions for 24-hour sedimentation at pH 6.5. (C) The normalized absorbance curve of 30 mg/L C_{60} suspension (for QCM-D experiments) for 24 hour sedimentation, and the absorbance at initial 120 minutes is enlarged. (D) The hydrodynamic diameters of 30 mg/L C_{60} suspension keep stable for a while after 30-min sonication.

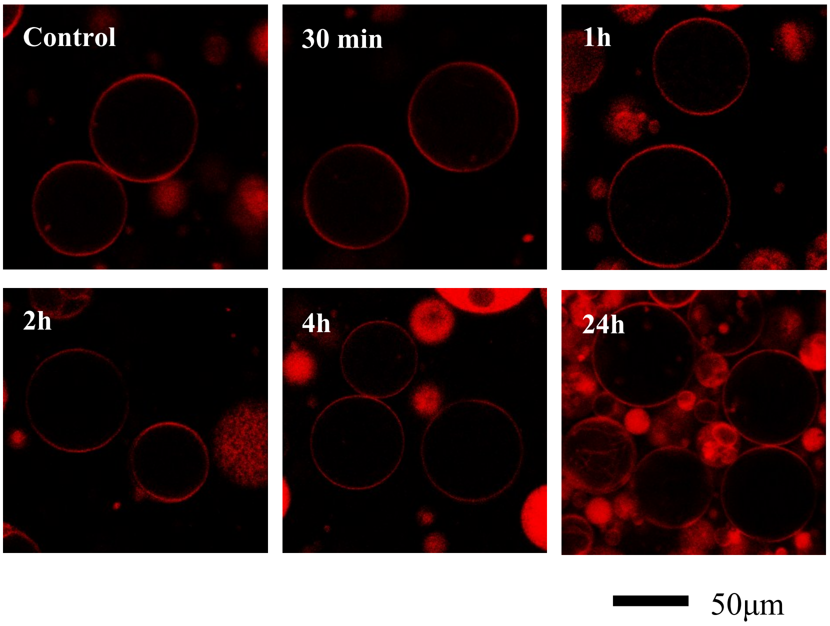

Fig. S2. Images of RhB-labeled GUVs^{-} after exposure to C_{60} NPs in 0.1 *M* glucose.

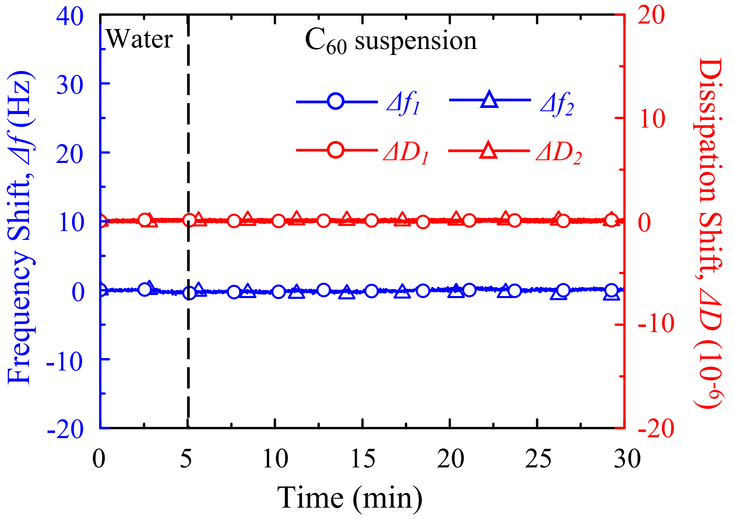

Fig. S3. The frequency shifts (Δf) and dissipation shifts (ΔD) when C_{60} NPs was directly injected into the measurement chamber without the formation of SLBs or SUVs. Δf_1 and ΔD_1 represent the results of silica-coated crystal sensor, and Δf_2 and ΔD_2 represent the results of gold crystal sensor. Both Δf and ΔD keep stable, indicating that C_{60} NPs do not deposit on gold or silica-coated crystal sensors.

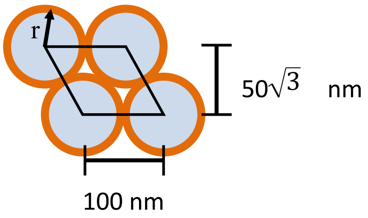

close packing
(PSD=0.907)

$$\text{Plane stacking density (PSD)} = \frac{\pi r^2}{area\ of\ quadrangle\ cell}$$

Fig. S4. Hypothetical arrangements of SUVs on 0.78 cm^2 sensor, surface area of SLB on sensor is regarded as 0.78 cm^2; surface area of the SUV layer on sensor is calculated to be 1.42 cm^2 in close packing. The ratio of the surface area of SUV layer to the surface area of SLB (A_{SUV}/A_{SLB}) is 1.814 in close packing.

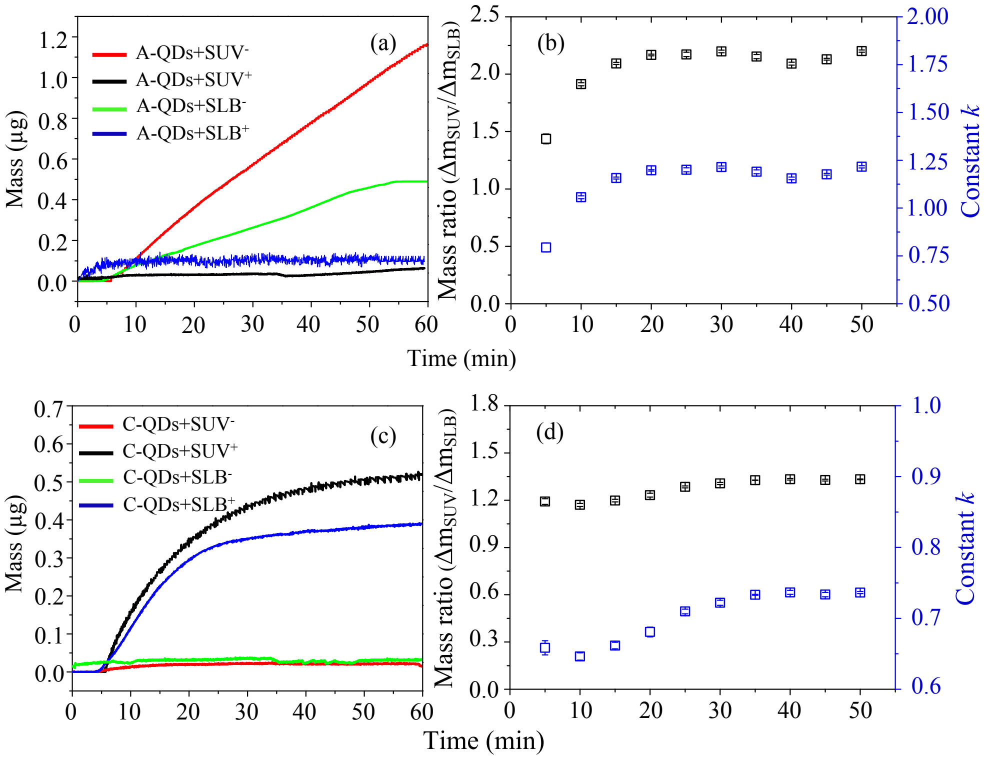

Fig. S5. The calculation of ratios of adhered mass of CdTe quantum dots (QDs) on SUV^- to the adhered mass on SLB^- ($\Delta m_{SUV}/\Delta m_{SLB}$) and the constant k using the published data in the literature.[3] (a) Mass changes on the SUV- and SLB-coated crystal sensors (0.78 cm^2) when amino-coated QDs (A-QDs) are injected into the QCM chamber. (b) The $\Delta m_{SUV}/\Delta m_{SLB}$ and the constant k are calculated as a function of time. (c) Mass changes when carboxyl-coated QDs (C-QDs) are injected into QCM chamber. (d) The $\Delta m_{SUV}/\Delta m_{SLB}$ and the constant k of C-QD adhesion are presented as a function of time.

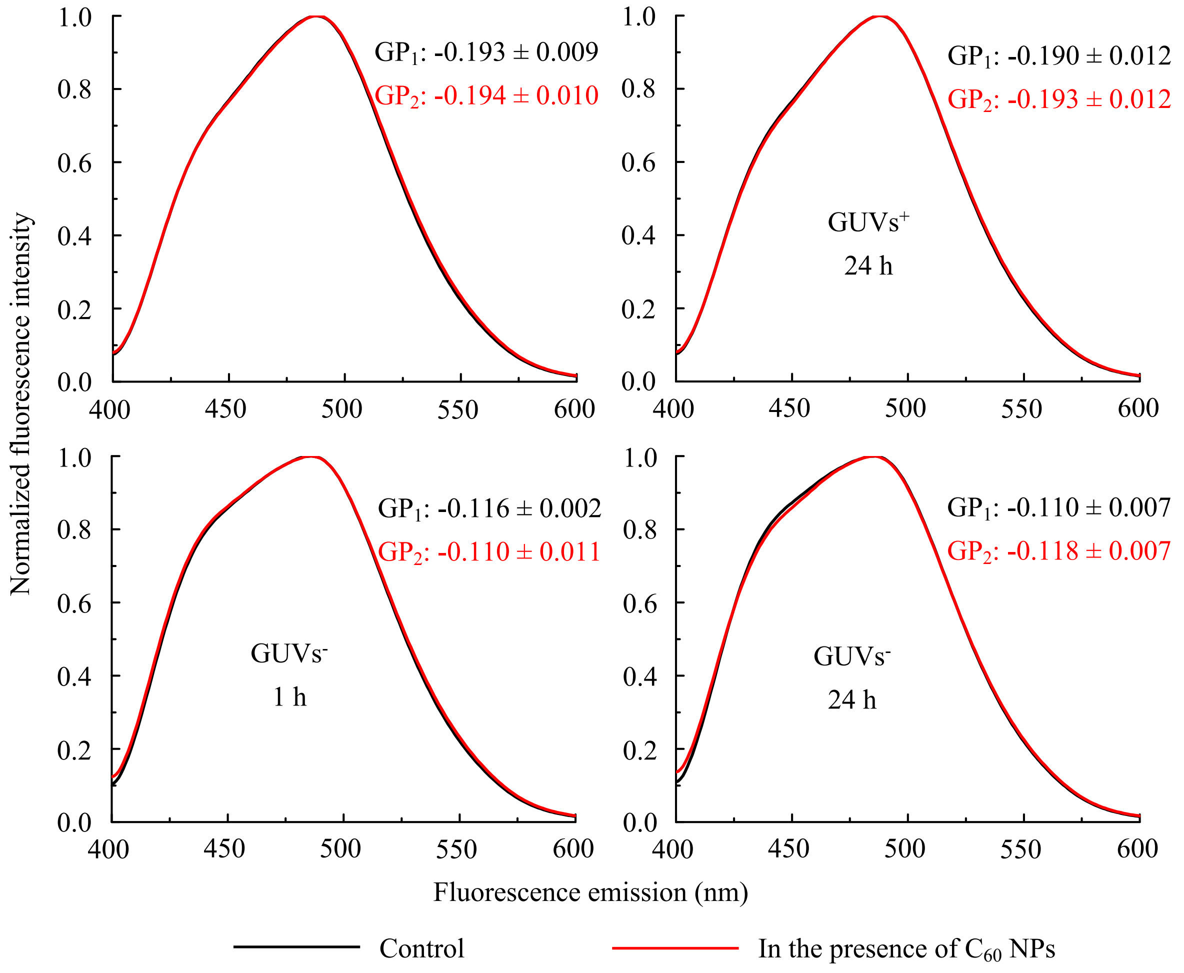

Fig. S6. Fluorescence spectra of Laurdan-labeled vesicles in the absence or in the presence of C_{60} NPs. GP_1 and GP_2 are the calculated GP values for control vesicles and the vesicles exposed to C_{60} NPs, respectively.

References

1 E. Reimhult, F. Höök and B. Kasemo, *Langmuir*, 2003, **19**, 1681-1691.

2 M. Yan, C. Liu, D. Wang, J. Ni and J. Cheng, *Langmuir*, 2011, **27**, 9860-9865.

3 M. Zhang, X. Wei, L. Ding, J. Hu and W. Jiang, *Environ. Pollut.*, 2017, **225**, 419-427.
